# Open Field Study of Some *Zea mays* Hybrids, Lipid Compounds and Fumonisins Accumulation

**DOI:** 10.3390/toxins7093657

**Published:** 2015-09-11

**Authors:** Paola Giorni, Chiara Dall’Asta, Massimo Reverberi, Valeria Scala, Matteo Ludovici, Martina Cirlini, Gianni Galaverna, Corrado Fanelli, Paola Battilani

**Affiliations:** 1 Dipartimento di Scienze delle Produzioni Vegetali Sostenibili, Università Cattolica del Sacro Cuore, Piacenza 29100, Italy; E-Mails: paola.giorni@unicatt.it (P.G.); paola.battilani@unicatt.it (P.B.); 2 Dipartimento di Scienze degli Alimenti, Università degli Studi di Parma, Parco Area delle Scienze 17/A, Parma 43124, Italy; E-Mails: chiara.dallasta@unipr.it (C.D.); martina.cirlini@unipr.it (M.C.); gianni.galaverna@unipr.it (G.G.); 3 Dipartimento di Biologia Ambientale, Università Sapienza, P. le Aldo Moro 5, Roma 00185, Italy; E-Mails: massimo.reverberi@uniroma1.it (M.R.); matteo.ludovici@uniroma1.it (M.L.); corrado.fanelli@uniroma1.it (C.F.)

**Keywords:** maize, *Fusarium verticillioides*, sphingolipids, oxylipins, mycotoxin

## Abstract

Lipid molecules are increasingly recognized as signals exchanged by organisms interacting in pathogenic and/or symbiotic ways. Some classes of lipids actively determine the fate of the interactions. Host cuticle/cell wall/membrane components such as sphingolipids and oxylipins may contribute to determining the fate of host–pathogen interactions. In the present field study, we considered the relationship between specific sphingolipids and oxylipins of different hybrids of *Zea mays* and fumonisin by *F. verticillioides*, sampling ears at different growth stages from early dough to fully ripe. The amount of total and free fumonisin differed significantly between hybrids and increased significantly with maize ripening. Oxylipins and phytoceramides changed significantly within the hybrids and decreased with kernel maturation, starting from physiological maturity. Although the correlation between fumonisin accumulation and plant lipid profile is certain, the data collected so far cannot define a cause-effect relationship but open up new perspectives. Therefore, the question—“Does fumonisin alter plant lipidome or does plant lipidome modulate fumonisin accumulation?”—is still open.

## 1. Introduction

Maize is a globally-cultivated crop destined to food and feed production. However, maize is also highly prone to fungal contamination and specifically to mycotoxigenic fungi [1]. Among the fungal pathogens that infect maize, *Fusarium verticillioides* Sacc. (Nirenberg) is frequently the dominant toxigenic fungus found in temperate areas. In addition to causing seedling blight, root rot, stalk rot, kernel rot, or ear rot during maize colonisation, *F. verticillioides* produce fumonisins, toxic secondary metabolites causing a large variety of toxicological effects on animals and humans. Fumonisins are able to disrupt sphingoid based metabolism, and are suspected of inducing nervous system diseases in animals and humans [2,3]. Fumonisin B_1_ (FB_1_) has been included in the class 2B by the International Agency for Research on Cancer, because of its possible carcinogenic effect in humans [3]. In 2007, the European Commission defined thresholds for FB_1_ + FB_2_ content in raw maize and its derived products intended for human consumption (EU Commission Regulation No. 1126/2007). Recommendations have also been defined for animal feeding with differences between species (EU Commission Recommendation No. 576/2006) to prevent animal diseases like equine leukoencephalomalacia or acute pulmonary oedema in swine. Among the fumonisins synthesized by *F. verticillioides*, FB_1_, FB_2_ and recently FB_3_ are reported as the dominant ones in maize [4,5]; the presence of modified forms of B series fumonisins in raw maize, not detected by analytical methods commonly applied, was also reported [6,7].

Several studies explored the factors affecting mycotoxin synthesis and notably fumonisins. Recent studies have shown that the chemical composition of maize kernels can influence fumonisin content [8,9,10]; *i.e.*, the composition of total fatty acids related closely to fumonisin accumulation in maize under field conditions. Notably, maize hybrids with high linoleic content displayed higher fumonisin contamination [8]. In general, lipid molecules are gaining *momentum* as crucial signals able to determine the fate of the interaction between cereals and mycotoxin-producing fungi [8,9,10,11]. In the plant cell, a particular class of lipid molecules, the sphingolipids (SL), are present in the cell membranes of all eukaryotes and play an important role during the interactions with the fungal pathogens [12]; ceramide (Cer), sphingosine (Sph), and sphingosine-1-phosphate (S1P) are part of this complex family of compounds. SL mediate different processes and a sharp modulation between Cer and S1P was highlighted. Notably, as the amount of Sph increases, the cell can initiate apoptosis or programmed cell death [13,14], whereas increases in S1P promote cell survival and proliferation [15]. In plants, SL concur to form the first line of defense against abiotic and biotic stresses. In particular, SL pre-cursors, *i.e.*, long chain bases (LCB), increase upon pathogen infection [16]. Further, they are potentially cytotoxic for pathogenic microorganisms. As reported [17], fumonisins are potent inhibitors of ceramide synthases, the enzyme involved in sphingolipids synthesis either *de novo* or through salvage pathway. Persistent fumonisin inhibition of Cer biosynthesis resulted in the production of toxic free sphingoid bases, increases in sphingoid base 1-phosphates, depletion of critical membrane glycosphingolipids, and altered phospholipid signaling pathways in animals and in maize cells and seedlings [18,19].

Dall’Asta *et al.* 2015 [9] demonstrated by an untargeted lipidomic approach that four lipid entities might differentiate *Zea mays* hybrids highly contaminated with fumonisins (HFb) by the low-contaminated ones (LFb). In this paper, we have tested the reliability of these markers by planning an open field experiment on a subset of HFb hybrids. Notably, we considered the relationship between specific sphingolipids and fumonisin by *F. verticillioides*, sampling ears of different hybrids of *Zea mays* from early dough to fully ripe in open field.

## 2. Results

### 2.1. Incidence of Fungal Species, F. verticillioides and Fumonisin Production

Fungi were detected in all growth stages of kernel sampling, with an increasing trend towards harvest time. Contamination by *Aspergillus* spp. was found to be very limited (3% as mean of all samples) and *Penicillium* spp. was detected only sporadically, mainly at harvest (Table 1).

Nevertheless, *Aspergillus* infection was limited. In some samples where it was present, no *Fusarium* strain was isolated (data not shown).

**Table 1:** Results of the analysis of variance (ANOVA) and Tuckey test. The factors considered were: hybrid and growth stage (GS) (see Methods Section) and the variables were the incidence of kernels infected by fungi (Fungi), *Aspergillus* spp. (AI) and *Fusarium* spp. (FI), Free (FFB) and total (HFB) fumonisins B (B1 + B2 + B3) and oxylipins and ceramides detected in maize samples: 9-HODE, 13-HODE, *N*-(2ʹ-hydroxylignoceroyl)-phytosphingosine (HLP), *N*-lignoceroyl-phytosphingosine (LP).

Factors	Fungi	AI	FI	FFB	TFB	9-HODE	13-HODE	HLP	PHY-CER	NOH
Hybrid	n.s.	n.s.	n.s.	**	*	**	*	**	**	**
H17	47.0	4.7	24.9	4109.2 ^b^	4682.8 ^b^	7.4 ^ab^	10.9	2.3 ^ab^	2.7 ^ab^	2.3 ^b^
H18	43.1	2.7	12.9	930.3 ^a^	531.8 ^a^	10.9 ^b^	11.3	2.9 ^b^	3.1 ^b^	2.8 ^b^
H19	40.6	1.6	16.3	588.8 ^a^	411.1 ^a^	6.4 ^a^	8.6	1.9 ^a^	1.7 ^a^	1.3 ^a^
H20	55.6	3.4	24.1	1943.2 ^ab^	2709.3 ^ab^	8.3 ^ab^	9.0	2.8 ^b^	1.8 ^a^	1.6 ^a^
Sampling Time	**	n.s.	**	**	**	n.s.	**	**	**	**
1	16.2 ^a^	7.9	7.4 ^a^	0.0 ^a^	0.0 ^a^	7.4	12.7 ^b^	2.8 ^b^	2.8 ^b^	2.9 ^b^
2	33.9 ^ab^	0.6	12.8 ^a^	481.4 ^a^	571.8 ^b^	9.7	9.8 ^ab^	2.8 ^b^	3.0 ^b^	2.4 ^b^
3	50.2 ^b^	3.9	16.4 ^a^	1690.6 ^b^	1734.7 ^b^	9.5	9.5 ^a^	2.2 ^ab^	2.4 ^b^	1.6 ^a^
4	85.9 ^c^	0.0	41.6 ^b^	5399.4 ^c^	6028.6 ^b^	6.4	7.8 ^a^	1.9 ^a^	1.1 ^a^	1.1 ^a^

n.s.: not significant; * *p* ≤ 0.05; ** *p* ≤ 0.01. ^a,b,c,ab^ different letters indicate significant differences among values.

**Figure 1:**
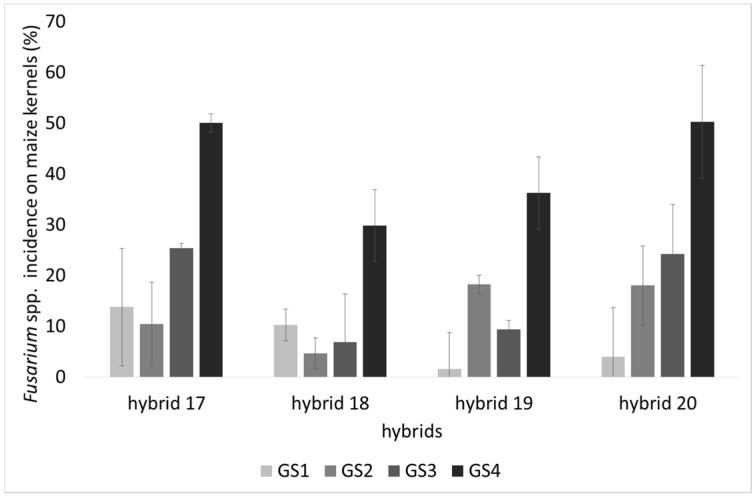
*Fusarium* spp. incidence (%) in naturally contaminated maize kernels from four commercial hybrids (17–20) harvested at four different growth stages: at early dough maturity (GS1), dough stage (GS2), physiological maturity (GS3) and fully ripe (GS4) in three locations in Italy. Results represent the mean of *n* = 9 incidence values deriving from the three different locations (biological replicates) in three technical replicates ± SE.

The incidence of kernels infected by *Fusarium* spp. significantly increased during the four growth stages (*p* < 0.001; Table 1). Notably, at GS4, hybrid 17 was found to be by far the most contaminated and hybrid 18 the least contaminated by *Fusarium* spp. (Mann Whitney Test; *p* < 0.01) (Figure 1).

**Figure 2:**
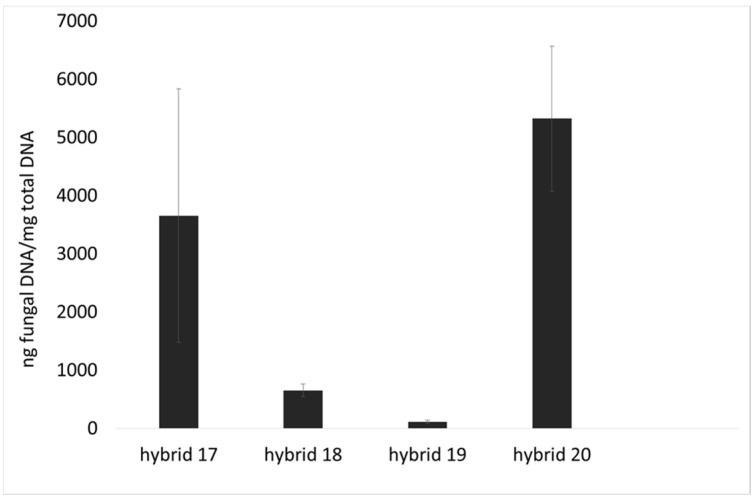
Abundance of fungal DNA (ng fungal DNA/μg total DNA) as a measure of *F. verticillioides* colonization of maize ears at fully ripe stage (GS4) from four commercial hybrids (17–20) cultivated in three locations in Italy. Results represent the mean of *n* = 6 DNA amount values deriving from the three different locations (biological replicates) in two technical replicates ± SE.

The amount of *F. verticillioides* DNA was measured at the fully ripe stage (GS4) by qPCR assay. A lower amount of *F. verticillioides* DNA was found in hybrids 18 and 19 compared to hybrid 17 (*p* < 0.05) and hybrid 20 (*p* < 0.001; Figure 2), in agreement with incidence data (Figure 1).

**Figure 3:**
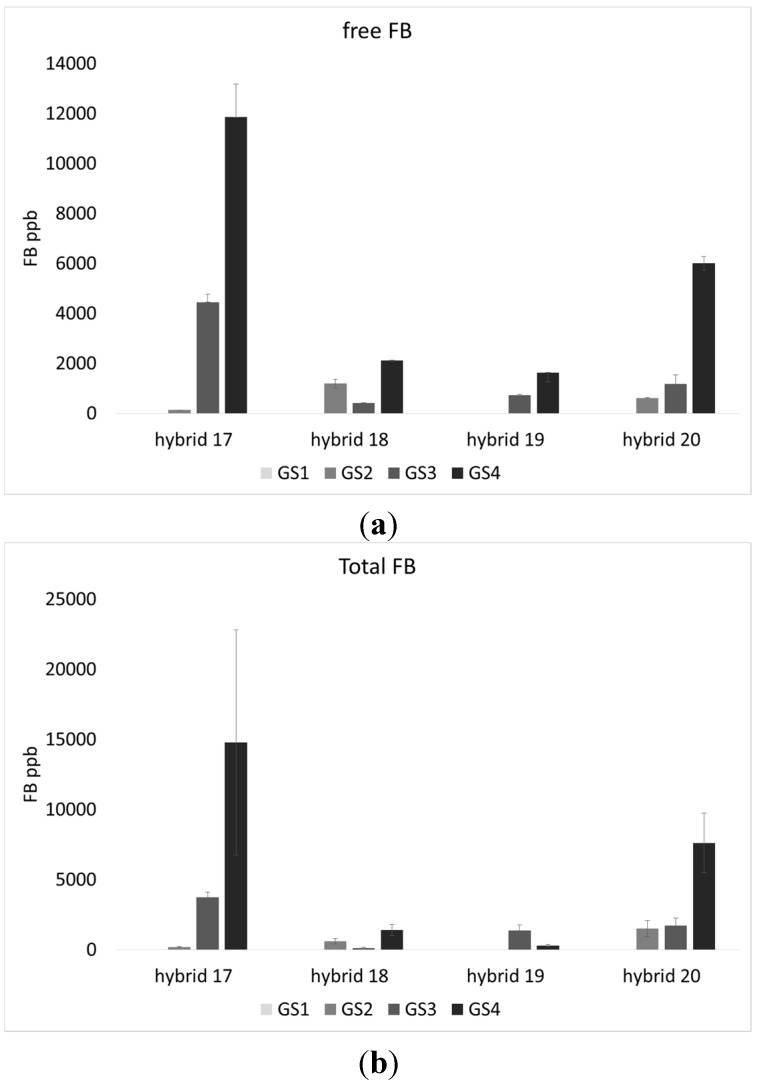
(**a**) Free; and (**b**) Hidden fumonisins (FB = FB1 + 2 + 3) content (ppb) in four maize hybrids, cultivated in three different localities and harvested at four different growth stages (GS1–4). Results represent the mean of *n* = 9 FB amount values deriving from the three different locations (biological replicates) in three technical replicates ± SE.

The total amount of fumonisin, in free and modified forms, differed significantly between hybrids and increased significantly with maize ripening. The lowest contamination was detected in hybrid 18 and 19, while hybrid 17 was the most contaminated both considering free and total fumonisins. It can be pinpointed that, as well as for fungal incidence, fumonisins, (a) free; and (b) total forms, increased in almost all the hybrids during the growth stages (Table 1; Figure 3a,b). The interaction between the hybrid and growth stage was not found to be significant for both fungi and fumonisin (data not shown).

### 2.2. Lipid Profile of Maize Challenged with F. verticillioides at the Field Level

**Figure 4:**
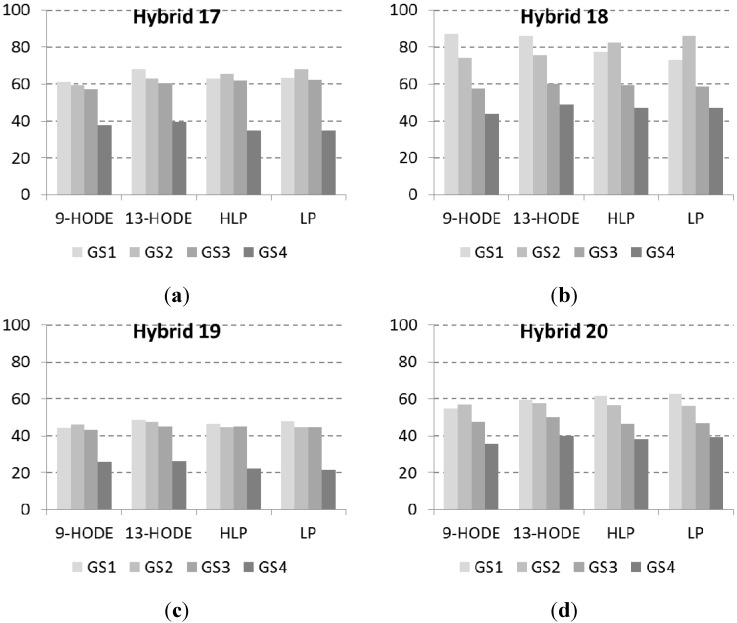
(**a**–**d**) *N*-(2ʹ-hydroxylignoceroyl)-phytosphingosine (HLP), *N*-lignoceroyl-phytosphingosine (LP), 9- and 13-HODE, expressed as percentage on the maximum amount detected in each growing location, in hybrid 17 (**a**); hybrid 18 (**b**); hybrid 19 (**c**) and hybrid 20 (**d**) harvested at different growth stages: early dough maturity (GS1), dough stage (GS2), physiological maturity (GS3) and fully ripe (GS4). Results represent the mean of *n* = 9 values deriving from the three different locations (biological replicates) in three technical replicates ± SE.

In relation to findings of [9], we focused our attention on the presence of specific fatty acid derivatives such as 9- and 13-HydroxyOctaDecEnoic acids (HODE), and phytoceramides such as lignoceric phytosphingosine (Cer(t18:0/24:0)) (LP) and hydroxyl-lignoceric phytosphingosine (Cer(t18:0/24:0(2OH))) (HLP) in the four commercial hybrids of maize naturally infected by *F. verticillioides*. 9-HODE and phytoceramides were significantly affected by hybrids, with hybrid 19 having always the lowest and hybrid 18 always the highest content. Fatty acid derivatives and phytoceramides decreased during kernel maturation. Specifically, the decrease started from the physiological maturity stage in hybrid 18 and hybrid 20, whilst it started from the fully ripe stage in hybrid 17 and hybrid 19 (Figure 4a–d), whereas 9-HODE was found to be unaffected by the growth stage (Table 2).

### 2.3. Correlations

The Pearson correlation was applied as a measure of the correlation between variables: oxylipins and ceramides detected in maize samples: 9-HODE, 13-HODE, HLP, LP, free (FFB) and total (TFB) fumonisins and the percentage of *Fusarium* infection in maize ears (*FI*). Pearson coefficients calculated from the whole dataset (“All Hybrids” = 48 observations) are shown in Table 2. The production of fumonisins (free forms), as well as fungal contamination, are negatively correlated with 13-HODE, HLP, LP biosynthesis.

**Table 2:** Pearson correlation coefficients amongst the variables in four hybrids of maize cultivated in three different Italian locations calculated on 48 observations. The variables considered are growth stage (GS) (see Methods Section), oxylipins and ceramides detected in maize samples: 9-HODE, 13-HODE, *N*-(2ʹ-hydroxylignoceroyl)-phytosphingosine (HLP), *N*-lignoceroyl-phytosphingosine (LP), free (FFB) and Total (TFB) fumonisins B (B1 + B2 + B3) and the percentage of *Fusarium* infection on maize ears (*FI*).

Variable	13-HODE	HLP	LP	FFB	TFB	*FI*
9-HODE	0.371 ^c^	0.482 ^c^	0.205 ^a^	−0.095	−0.082	−0.130
13-HODE		0.683 ^c^	0.715 ^c^	−0.208 ^a^	−0.107	−0.381 ^c^
HLP			0.805 ^c^	−0.221 ^a^	−0.185	−0.413 ^c^
LP				−0.226 ^a^	−0.179	−0.416 ^c^
TFB					0.976 ^c^	0.491 ^c^
HFB						0.309 ^b^

^a^: *p* < 0.05; ^b^: *p* < 0.01; ^c^: *p* < 0.001.

## 3. Discussion

Metabolite profiling under field conditions is becoming increasingly popular. Several biotic and abiotic stresses affect plant metabolomes, which is rapidly becoming a central topic in metabolite-profiling experiments in the field [20]. The environment may buffer different stresses [21,22] and, in relation to this, the alteration of the plant metabolic profile under field conditions is more suggestive of its modification occurring under *ex vivo* conditions, e.g., in phytochambers [23]. Dall’Asta *et al.* [9] indicated, by a lipidomic approach applied in an open field study, that some phytoceramides distinguish maize hybrids conducive for fumonisin synthesis from non-conducive ones. Thus, in the present study, we planned open field experiments in three areas of Northern Italy, for evaluating the trend of these lipid markers in four different hybrids and the correlation with fumonisin accumulation.

As expected, almost all the lipid markers varied significantly between hybrids. In particular, in hybrid 19, the lowest amount was always detected, and, as such, so was the lowest fumonisin contamination. Besides, FFB were significantly and negatively correlated to 13-HODE, HLP and LP. This confirms the correlation of lipid derivatives’ content, including phytoceramides, in maize hybrids and fumonisin production. The few field studies, therefore, highlighted the role of plants in the regulation of fumonisin production under natural field conditions.

Fumonisins are inhibitor of ceramide synthases, enzymes which, starting from sphingosine, sphinganine and fatty acids, produce ceramides and, in plant, phytoceramides [24]. The inhibition of ceramide synthase alter the sphingolipid metabolism that, in turn, could trigger apoptosis, as demonstrated in mammalian [25].

Novel insights, deriving from genetic and biochemical studies, indicate that free sphingosines, as well as ceramides and their respective phosphorylated forms, play a critical role in the regulation of plant defence [15].

In mammals, bacterial and viral pathogens target predominantly structural and signalling lipids to alter the cellular phenotype of the host cell. On the contrary, fungal pathogens have complex lipidomes themselves and target predominantly the release of polyunsaturated fatty acids from the host cell lipidome. Authors reported that “eukaryotic pathogens focus on interference with lipid metabolites affecting systemic inflammatory reactions that are part of the host immune system” [26].

Desjardins *et al.* [27] correlated fumonisin-induced diseases in animals with the biochemical consequences of ceramide synthase inhibition. Over recent years, Proctor’s group indicated that fumonisin production by *F. verticillioides* is required for developing full symptoms of foliar disease in maize seedlings [19]. Moreover, these authors suggested, “the overall incidence and severity of seedling disease development are likely dependent on both maize genotype and the amount of fumonisin produced by *F. verticillioides* strains”. According to Williams *et al.* [2], fumonisins cause an important cascade of events leading to altered cell growth, differentiation, and cell injury. These events occurred *in vitro* as well as *in vivo.* Therefore, these authors propose that a prolonged exposure of maize plant to fumonisins resulted in a change of expression or relative activity of the enzymes responsible for metabolizing free sphingoid bases. Sanchez-Rangel *et al.* [28] further confirm the involvement of fumonisin production in fungal virulence showing that *F. verticillioides* and exogenously added FB_1_ down-modulate maize β-1,3-glucanases (PR2-like proteins). In *Arabidopsis*, FB_1_ might trigger plant defense hallmarks such as ROS production, phenolics and callose deposition, biosynthesis of phytoalexins, plus the expression of PR proteins and the activation of the jasmonate/ethylene and SA-dependent signaling pathways also related to PCD (Programmed Cell Death) onset [15]. Berkey *et al.* [15] propose that the sphinganine-like toxins, such as FB_1_, may induce PCD in plant by inhibiting acyl-CoA-dependent ceramide synthases. The production of fumonisins may result as part of a strategy exploited during the necrotrophic phase of *F. verticillioides* and suggests a close relation between sphingolipid metabolism and plant PCD. Regarding this, *F. verticillioides* could foster mycotoxin accumulation in maize, profoundly altering the sphingolipid metabolism.

Starting from these evidences, we used field studies for verifying at real scale the correlation between the fumonisins’ biosynthesis by *F. verticilllioides* and the synthesis of some phytoceramides during its interaction with the host. Our data show a negative correlation between lignoceric acid sphingosine and its hydroxylated forms, and the amount of fumonisin and fungal infection. These results are confirmed in three different growing locations, which provides consistency to the output.

The observed decrease in these ceramides could cause an increase in their related phytosphingosine moiety (t18:0). Several studies report that a rapid accumulation of endogenous dihydrosphingosine (d18:0) and t18:0 occurs within hours after FB_1_ treatment. This increase in d/t18:0 was correlated with PCD onset in plants as well as in animals [15].

In our study, we may hypothesise that in the hybrids conducive to fumonisins, the ceramide synthases are affected and a decrease in the amount of two specific phytoceramides occurs. This event could lead to an increase in t18:0 which, in turn, favours fungal growth inducing PCD, as stated by other authors [15]. We also confirm a close relationship between the whole lipid profile of maize kernels and fumonisin accumulation [8,9]. In relation to this, we also proved in this study that 13-HODE, a LOX-oxylipin, decreases significantly during the growing season, *i.e.* with the increase of fungal infection and fumonisin production. Thus, the ability of this pathogen to grow on maize ears is probably related with the reduction of this defense oxylipin.

Although fumonisin accumulation and the plant lipid profile are correlated, the data collected so far cannot define a cause–effect relationship. Therefore, the question—“Does fumonisin alter the plant lipidome or does the plant lipidome modulate fumonisin accumulation?”—is still open.

In conclusion, our data shows that, as suggested by Glenn *et al.*, [19], both maize genotype and fumonisin play a role in the disease, but the role of genotype seems to be more important under field conditions.

## 4. Materials and Methods

### 4.1. Maize Sample Collection

Samples from four commercial hybrids were collected during the growing season in diverse locations of Northern Italy: Cremona, Pordenone and Torino. Sampling was carried out every two weeks, from maize early dough stage up to harvest time, therefore four times per field (growth stages 1–4; GS) indicatively, following the BBCH-identification keys of maize, corresponding to BBCH83 early dough (GS1), BBCH85 dough (GS2), BBCH87 physiological maturity (GS3), BBCH 89 fully ripe (GS4) [29,30].

Hybrids belong to different FAO classes: FAO 500 (one hybrid) and FAO 600 (three hybrids). The hybrid names were not reported because only a limited number of hybrids were considered and because the aim was to find out relevant factors not related to a specific hybrid, but of more general value, having in mind the short commercial life of hybrids. The used hybrids, commercially available in Italy, were reported with increasing numbers from hybrid 17 to 20 according to sample codification used in previous studies [8,9]. Ten ears were collected from three different rows chosen longitudinally along the field area; 30 ears were sampled for each hybrid. After husk elimination, ears were shelled and kernels of each sample were shared in two sub-samples of about 1 kg used for mycological and chemical-molecular analysis, respectively. Samples destined to chemical and molecular analysis were stored at −20 °C while the others were immediately processed.

### 4.2. Incidence of Infected Kernels

Fifty kernels were randomly selected from each sample and surface disinfected in 1% sodium hypochlorite solution for 2 min and then in 90% ethyl alcohol solution for 2 min. Kernels were rinsed with sterile distilled water and dried under a sterile hood. Grain kernels were plated on Petri dishes (Ø 9 cm) containing Potato Dextrose Agar (PDA, Oxoid Ltd., Basingstoke, Hampshire, UK) added with 0.1% streptomycin (Sigma-Aldrich, St. Louis, MO, USA) and incubated at 25 °C for 7 days with a 12 h light photoperiod. After incubation, kernels showing mould development were counted. The identification of growing colonies at genus level was done for *Fusarium*, *Aspergillus* and *Penicillium* [31,32,33]. Data obtained were used to calculate the incidence of fungi and of the above-mentioned genera on plated kernels.

### 4.3. Fungal Growth by qPCR

In order to monitor fungal growth in maize cobs, a specific SYBR green qPCR method was set by using *FUM1* primers [10]. Real-time PCR was prepared in a 20 μL reaction mixture as described in [10].

### 4.4. Chemicals

Authentic oxylipins 9-hydroxy-10E,12Z-octadecadienoic acid (9-HODEd4, MW 300.5) and 13-hydroxy-9*Z*,11*E*-octadecadienoic acid (13-HODEd4, MW 300.5) were purchased from Cayman Chemicals (Ann Arbor, MI, USA) whereas authentic sphingolipids, *N*-(2ʹ-hydroxylignoceroyl)-phytosphingosine (Cer (t18:0/24:0(2OH), MW 683.6)) and *N*-lignoceroyl-phytosphingosine (Cer (t18:0/24:0), MW 667.6)) and *N*-palmitoyl-d31-d-*erythro*-sphingosine (C16-D31 Ceramide, MW 568.7) were purchased from Avanti polar lipids, Inc. (Alabaster, AL, USA).

### 4.5. Lipid Analysis

Lipid extraction and chromatography conditions were selected according to [10]. Deuterated compounds, such as 9-HODE d4, 13-HODE d4, and C16-d31 ceramide were used as the internal standards (ISTD). The amount of each ISTD added to the matrix before the extraction was 400 pmol, which corresponded to the final concentration of 2 µM when the dried extracts were dissolved in the final volume of 200 µL of MeOH. The analysis of lipid extracts was performed according to the previously reported method [10]. Briefly, lipid extracts were analyzed by rapid resolution reversed phase HPLC (RR-RP-HPLC) coupled with a triple quadrupole (QqQ) mass spectrometer (G6410A series triple quadrupole, QqQ, Agilent Technologies, Santa Clara, CA, USA) following ESI ionization as reported in [9] with slight modifications. In particular, negative and positive detection modes were selected for oxylipins and sphingolipids, respectively. In the first time segment, 0–3 min oxylipins were acquired in ESI negative ion mode, 3–38 min phytoceramides were acquired in ESI positive ion mode. Switch of polarity to positive ion mode to detect sphingolipids was set at 3 min. To acquire oxylipins and sphingolipids dynamic, multiple reaction monitoring (dMRM) experiments were performed [34]. The MRM transitions (Table 3), acquired by injecting authentic standards, were in agreement with the literature [33]. Relative abundance of each oxylipin (9 and 13-HODE), phytoceramide (*N*-(2ʹ-hydroxylignoceroyl)-phytosphingosine (HLP) and *N*-lignoceroyl-phytosphingosine (LP)) was expressed as the ratio between the compound and the ISTD transition. MRM data were processed using Mass Hunter Quantitative software (B.03.02 version, Agilent Technologies, Santa Clara, CA, USA).

**Table 3:** Mass transitions, collision energy (CE), fragmentor energy and retention times of the lipid entities analyzed.

Species	Precursor/Product Ion *m*/*z*	CE (eV)	Fragmentor (eV)	RT (min)
9-HODE d4 (ISTD)	299.2–>172.2	−20	−140	1.22
9-HODE	295.2–>171.2	−20	−140	1.22
13-HODE d4 (ISTD)	299.2–>198.2	−20	−140	1.23
13-HODE	295.2–>195.2	−20	−140	1.23
C16-D31 Ceramide (ISTD)	568.6–>264.2	+30	+140	15.8
Cer (t18:0/24:0(2OH))	684.6–>282.2	+34	+140	17.95
Cer (t18:0/24:0)	668.6–>282.2	+34	+140	18.35

### 4.6. Free and Total Fumonisins

Free and total fumonisins were determined according to our previous works [6,7,8,9]; each of these papers add an additional step to the full method we used for this work. Fumonisins obtained after sample hydrolysis were measured as the sum of hydrolyzed FB_1_, FB_2_, and FB_3_. All of the results are expressed as the sum of FB_1_, FB_2_, and FB_3_ equivalents, considering a correction factor due to the different molecular weights of parent and hydrolyzed compounds, and referred to as “total fumonisins” (FB_tot_).

### 4.7. Statistics

The analysis of variance (ANOVA) was applied to define the role of hybrids and their growth stage on all the parameters considered using growing locations (*n* = 3) as replicates; means were compared using the Tukey test. Fumonisin and lipid compounds data were rated according to the maximum amount detected per growing area and arcsen transformed before statistical analysis.

Two-paired comparisons were performed by Mann-Whitney Test and relative significance (*p*) calculated accordingly. The relationships between some variables were also tested for linearity calculating the relevant Pearson (*p*) correlation coefficients.

Data analysis was managed with PASW statistics 21 (ver. 21, SPSS Inc., Chicago, IL, USA, 2012).

## Abbreviations

Cer	Ceramide
Free FB	Free fumonisins B
Hidden FB	Hidden fumonisins B
FB_1_	fumonisin B_1_
HODE	hydroxyoctadecenoic acids
MRM	multiple reaction monitoring
HLP	*N*-(2ʹ-hydroxylignoceroyl)-phytosphingosine
LP	*N*-lignoceroyl-phytosphingosine
qPCR	quantitative PCR
ROS	reactive oxygen species
Sph	sphingosine
S1P	sphingosine-1-phosphate
FB_tot_	total fumonisins
QqQ	triple quadrupole mass spectrometer

## References

[1] Woloshuk C.P., Shim W.B. (2013). Aflatoxins, fumonisins, and trichothecenes: A convergence of knowledge. FEMS Microbiol. Rev..

[2] Williams L.D., Glenn A.E., Zimeri A.M., Bacon C.W., Smith M.A., Riley R.T. (2007). Fumonisin disruption of ceramide biosynthesis in maize roots and the effects on plant development and *Fusarium verticillioides*-induced seedling disease. J. Agric. Food Chem..

[3] IARC (1993). Monographs on the Evaluation of Carcinogenic Risks to Humans, Beryllium, Cadmium, Mercury, and Exposures in the Glass Manufacturing Industry.

[4] Lazzaro I., Falavigna C., Dall’Asta C., Galaverna G., Battilani P. (2012). Fumonisins B, A and C profile and masking in *Fusarium verticillioides* strains on fumonisin-inducing and maize-based media. Int. J. Food Microbiol..

[5] Lazzaro I., Falavigna C., Galaverna G., Dall’Asta C., Battilani P. (2013). Cornmeal and starch influence the dynamic of fumonisin B, A and C production and masking in *Fusarium verticillioides* and *F. proliferatum*. Int. J. Food Microbiol..

[6] Dall’Asta C., Mangia M., Berthiller F., Molinelli A., Sulyok M., Schumacher R., Krska R., Galaverna G., Dossena A., Marchelli R. (2009). Difficulties in fumonisin determination: The issue of hidden fumonisins. Anal. Bioanal. Chem..

[7] Dall’Asta C., Falavigna C., Galaverna G., Dossena A., Marchelli R. (2010). *In vitro* digestion assay for determination of hidden fumonisins in maize. J. Agric. Food Chem..

[8] Dall’Asta C., Falavigna C., Galaverna G., Battilani P. (2012). Role of maize hybrids and their chemical composition in *Fusarium* infection, fumonisin production and masking. J. Agric. Food Chem..

[9] Dall’Asta C., Giorni P., Cirlini M., Reverberi M., Gregori R., Ludovici M., Camera E., Fanelli C., Battilani P., Scala V. (2014). Maize lipids play a pivotal role in the fumonisin accumulation. World Mycotoxin J..

[10] Scala V., Giorni P., Cirlini M., Ludovici M., Visentin I., Cardinale F., Fabbri A.A., Fanelli C., Reverberi M., Battilani P. (2014). LDS1-produced oxylipins are negative regulators of growth, conidiation and fumonisin synthesis in the fungal maize pathogen *Fusarium verticillioides*. Front. Microbiol..

[11] Christensen A.S., Nemchenko A., Park Y.S., Borrego E., Huang P.C., Schmelz E.A., Kunze S., Feussner I., Yalpani N., Meeley R. (2014). The novel monocot-specific 9-lipoxygenase ZmLOX12 is required to mount an effective jasmonate-mediated defense against *Fusarium verticillioides* in maize. Mol. Plant Microbe Interact..

[12] Konig S., Feussner K., Schwarz M., Kaever A., Iven T., Landesfeind M., Ternes P., Karlovsky P., Lipka V., Feussner I. (2012). Arabidopsis mutants of sphingolipid fatty acid a-hydroxylases accumulate ceramides and salicylates. New Phytol..

[13] Hannun Y.A., Obeid L.M. (2008). Principles of bioactive lipid signalling: Lessons from sphin-golipids. Nat. Rev. Mol. Cell Biol..

[14] Van Brocklyn J.R., Williams J.B. (2012). The control of the balance between ceramide and sphingosine-1-phosphate by sphingosine kinase: Oxidative stress and the seesaw of cell survival and death. Comp. Biochem. Physiol..

[15] Berkey R., Bendigeri D., Xiao S. (2012). Sphingolipids and plant defense/disease: The “death” connection and beyond. Front. Plant Sci..

[16] Guillas I., Puyaubert J., Baudouin E. (2013). Nitric oxide sphingolipid interplays in plant signaling: An enigma from the Sphinx?. Front. Plant Sci..

[17] Pewzner-Jung Y., Ben-Dor S., Futerman A.H. (2006). When do Lasses (longevity assurance genes) become CerS (ceramide synthases)? Insights into the regulation of ceramide synthesis. J. Biol. Chem..

[18] Zitomer N.C., Mitchell T., Voss K.A., Bondy G.S., Pruett S.T., Garnier-Amblard E.C., Liebeskind L.S., Park H.E., Wang M.C., Sullards A H. (2009). Ceramide Synthase Inhibition by Fumonisin B1 Causes Accumulation of 1-Deoxysphinganine. J. Biol. Chem..

[19] Glenn A.E., Zitomer N.C., Zimeri A.M., Williams L.D., Riley R.T., Proctor R.H. (2008). Transformation-mediated complementation of a FUM gene cluster deletion in *Fusarium verticillioides* restores both fumonisin production and pathogenicity on maize seedlings. Mol. Plant Microbe Interact..

[20] Guan X.L., Wenk M.R. (2006). Mass spectrometry-based profiling of phospholipids and sphingolipids in extracts from *Saccharomyces cerevisiae*. Yeast.

[21] Fester T. (2015). Plant metabolite profiles and the buffering capacities of ecosystems. Phytochemistry.

[22] Folke C., Carpenter S., Walker B., Scheffer M., Elmqvist T., Gunderson L., Holling C.S. (2004). Regime shifts, resilience, and biodiversity in ecosystem management. Ann. Rev. Ecol. Evol. Syst..

[23] Atanasova-Penichon V., Pons S., Pinson-Gadais L., Picot A., Marchegay G., Bonnin-Verdal M.N., Ducos C., Barreau C., Roucolle J., Sehabiague P. (2012). Chlorogenic acid and maize ear rot resistance: A dynamic study investigating *Fusarium graminearum* development, deoxynivalenol production, and phenolic acid accumulation. Mol. Plant Microbe Interact..

[24] Zitomer N.C., Riley R.T. (2011). Extraction and analysis of fumonisins and compounds indicative of fumonisin exposure in plant and mammalian tissues and cultured cells. Methods Mol. Biol..

[25] Wang H., Jones C., Ciacci-Zanella J., Holt T., Gilchrist D.G. (1996). Fumonisins and *Alternaria alternata* lycopersici toxins: Sphinganine analog mycotoxins induce apoptosis in monkey kidney cells. Proc. Natl. Acad. Sci. USA.

[26] Helms J.B., Kaloyanova D.V., Strating J.R.P., van Hellemond J.J., van der Schaar H.M., Tielens A.G.M., van Kuppeveld F.J.M., Brouwers J.F. (2015). Targeting of the hydrophobic metabolome by pathogens. Traffic.

[27] Desjardins A.E., Plattner R.D., Nelsen T.C., Leslie J.F. (1995). Genetic analysis of fumonisin production and virulence of Gibberella fujikuroi mating population A (*Fusarium moniliforme*) on maize (*Zea mays*) seedlings. Appl. Environ. Microbiol..

[28] Sanchez-Rangel D., Sanchez-Nieto S., Plasencia J. (2011). Fumonisin B1, a toxin produced by *Fusarium*
*verticillioides*, modulates maize beta-1,3-glucanase activities involved in defense response. Planta.

[29] Weber E., Bleiholder H. (1990). Explanations of the BBCH decimal codes for the growth stages of maize, rape, faba beans, sunflowers and peas—With illustrations. Gesunde Pflanzen.

[30] Lancashire P.D., Bleiholder H., Boom T., Langeluddeke P., Stauss R., Weber E., Witzenberger A. (1991). A uniform decimal code for growth stages of crops and weeds. Ann. Appl. Biol..

[31] Summerell B.A., Salleh B., Leslie J.F. (2003). A utilitarian approach to *Fusarium* identification. Plant Dis..

[32] Raper K.B., Fennell D.I., Robert E. (1965). The Genus Aspergillus.

[33] Pitt J. (1979). The Genus Penicillium.

[34] Ludovici M., Ialongo C., Reverberi M., Beccaccioli M., Scarpari M., Scala V. (2014). Quantitative profiling of oxylipins through comprehensive LC-MS/MS analysis of *Fusarium verticillioides* and maize kernels. Food Addit. Contam..

